# Evaluation of Hepatoprotective Activity of *Caralluma europaea* Stem Extract against CCl_4_-Induced Hepatic Damage in Wistar Rats

**DOI:** 10.1155/2021/8883040

**Published:** 2021-01-07

**Authors:** Hayat Ouassou, Mohamed Bouhrim, Nour Elhouda Daoudi, Hassane Mekhfi, Abderrahim Ziyyat, Abdelkhaleq Legssyer, Mohamed Aziz, Mohamed Bnouham

**Affiliations:** Laboratory of Bioresources Biotechnology Ethnopharmacology and Health, Mohammed First University, Oujda, Morocco

## Abstract

The present study aims to evaluate the hepatoprotective activity of stem aqueous extract of *Caralluma europaea* (AECe) on carbon tetrachloride- (CCl_4_-) induced hepatic damage in Wistar rats. The animals were daily treated with the aqueous extract of *C. europaea* at a dose of 250 mg/kg body weight for 14 days. CCl_4_ was injected (1 ml/kg, *i.p*.) two times, on the 7^th^ and 14^th^ days. At the end of the experimental period, all rats were anesthetized to collect blood for the assessment of biochemical parameters and then sacrificed to collect the liver for weighing. Hepatotoxicity was evaluated by measuring the serum levels of aspartate aminotransferase (AST), alanine aminotransferase (ALT), alkaline phosphatase (ALP), bilirubin (total and direct), malondialdehyde (MDA), total protein (TP), triglycerides (TG), total cholesterol, very low-density lipoprotein (VLDL-c ), low-density lipoprotein (LDL-c), high-density lipoprotein (HDL-c), urea, creatinine, and uric acid. Based on the results obtained in this study, the administration of *C. europaea* before exposure to the administration of CCl_4_ conferred favorable hepatoprotective effect in rats. The treatment with AECe (250 mg/kg) exhibits a significant hepatoprotective effect by ameliorating CCl_4_-induced alterations of these biochemical parameters. Hence, *C. europaea* could be a potential medicinal herb that can be used in the future to prevent liver intoxication.

## 1. Introduction

The liver is an interesting organ in the human body. It plays a vital role in the maintenance, performance, and regulating the homeostasis of the body [1]. It has a central role in detoxification and excretion of endogenous and exogenous substances [2]. The high incidence of liver damage is caused by drugs, alcohol consumption, and environmental chemicals/xenobiotics, which lead to liver diseases such as hepatitis [1, 3]. Most of the hepatotoxic chemicals produce liver cell damage by inducing an increase in tissue lipid peroxidation, oxidative stress, and serum levels of many biochemical markers such as transaminases, alkaline phosphatase, bilirubin, triglycerides, and cholesterol [3, 4].

Carbon tetrachloride- (CCl_4_-) induced liver injury is the best-characterized animal model of xenobiotic-induced free-radical-mediated hepatotoxicity. CCl_4_ is converted into two free radicals, which are trichloromethyl radical (CCl_3_) and proxy trichloromethyl radical (OOCCl_3_) by cytochrome P450 [5]. These free radicals are capable of initiating lipid peroxidation and liver damage [6]. Several studies indicate that antioxidants protect the liver from oxidative damage, and they can prevent the risk of liver diseases [7]. Therefore, much attention has been focused on natural antioxidants. Many studies have been shown that medicinal plants are very rich in antioxidant compounds that exhibited powerful hepatoprotective activity by improving antioxidant status [8]. Among many medicinal plants*, Caralluma europaea* (CE) is one of the medicinal species belonging to the Apocynaceae family. It is widely distributed in Morocco, Algeria, Egypt, Spain, and Italy [9]. *C. europaea* has been traditionally used in the treatment of different diseases such as diabetes, cancer, cyst, kidney stones, and respiratory and cardiovascular disorders [10–13]. The juicy stems of *Caralluma europaea* are consumed as food [10, 14]. Besides, the stems of *C. europaea* are orally taken with water or milk to treat diabetes. Also, the stems roasted are administered with garlic and tomato as an antidiabetic salad [10]. Several pharmacological reports have confirmed the antioxidant, antimicrobial, antiproliferative, antidiabetic, and anti-inflammatory activities of *C. europaea* [15–19]. Moreover, previous studies have been reported on the biological activities of extracts obtained from many species of *Caralluma* such as hepatoprotective [1], anti-inflammatory [20], antidiabetic [21], antioxidant [22], and cytotoxic [23] activities. To the best of our knowledge, no work on the hepatoprotective activity of *C. europaea* stems has been reported to date, which encourage us to investigate the hepatoprotective effect of stem aqueous extract of *C. europaea* (AECe) against CCl_4_-induced hepatic damage in rats.

## 2. Materials and Methods

### 2.1. Plant Material

The fresh plant was bought from the herb market. It is authenticated botanical by the expert botanist Mohammed Fennan from the scientific institute of the University Mohammed V., and the specimen was deposited under the voucher number HUMPOM 150 in the herbarium at University Mohammed I., Oujda (Morocco), for future reference.

### 2.2. Preparation of Plant Extract

The stem parts were cut into small pieces and dried. After complete drying, the dried plant material was powdered by a mechanical grinder. The powdered plant (200 g) material was then extracted with 800 mL of distilled water. The whole mixture was macerated overnight and filtered. The filtrate was evaporated to eliminate water and to obtain the extract in dried form. A fresh solution was prepared from the dried residue in each day of treatment.

### 2.3. Chemicals Used

The following drugs and reagents were used in this study: Carbon tetrachloride (CCl_4_) and silymarin were purchased from Sigma-Aldrich, USA, standard Kits for assay of aspartate transaminase (AST), alanine transaminase (ALT), alkaline phosphatase (ALP), Total cholesterol, triglycerides, Glucose, High-density lipoprotein (HDL-c), Bilirubin (total and direct), Creatinine, Urea, and uric acid levels were purchased from Biosystems, Spain, and Diethyl ether was obtained from (Sigma-Aldrich, Germany).

### 2.4. Experimental Animals

Thirty healthy adult Wistar rats (♂/♀ = 1) weighing between 150–200 g were used in this study. The animals were taken from the animal house of the Faculty of Sciences, Mohammed First University, Oujda, Morocco. The rats were housed in polypropylene cages in a well-ventilated room with soft bedding and accessibility to water and food *ad libitum* in an environmentally controlled room (23 ± 2°C, 12 h dark/12 h bright). The rats were adapted one week preceding treatment.

All rats have cared in compliance with the internationally accepted Guide for the Care and Use of Laboratory Animals, published by the US National Institutes of Health (NIH publication no. 85-23, revised in 1985) [24].

### 2.5. Preparation of Doses and Treatments

The CCl_4_ was administered at a dose of 1 mL/kg (*i.p*.) with vehicle (olive oil) [25]. The aqueous extract of *C. europaea* was administered at a single dose of 250 mg/kg. The dose of the aqueous extract of *C. europaea* (250 mg/kg) was selected based on the previous efficacy studies (acute and subacute toxicity studies and many previous pharmacological activities of *C. europaea*) [19, 26]. Silymarin (40 mg/kg) was administered to the animals orally [27].

### 2.6. CCl_4_-Induced Hepatotoxicity Model in Rats

**Figure 1:**
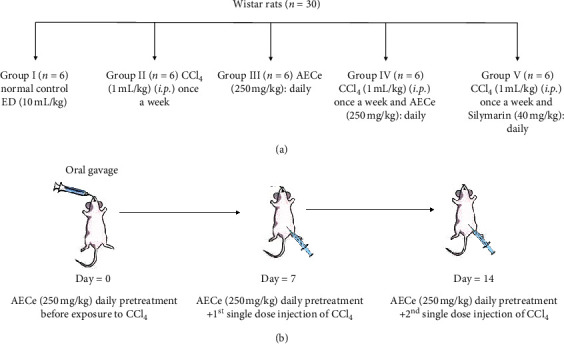
Schematic representation of (a) the experimental design; (b) the timeline chart for AECe treatment in the experimental rats injected with CCl_4_ (Group IV). n: number of rats.

One week after the adaptation, the animals were divided into five experimental groups of 6 animals each (♂/♀ = 1 : 3 males and 3 females), and treated as follows: the normal control received distilled water (10 mL/kg). The CCl_4_-treated control group (negative control) received distilled water (10 mL/kg). The AECe and AECe + CCl_4_ groups received a single dose of the aqueous extract (250 mg/kg). The positive group received CCl_4_ + silymarin (40 mg/kg). The animals of the CCl_4_-treated control group, AECe + CCl_4_ group, and CCl_4_ + silymarin group received CCl_4_ intraperitoneally (*i*.*p*.) at a dose 1 mL/kg body weight once a week for two weeks (the 7^th^ and 14^th^ days) of treatment in order to induce chronic liver injury. All animals were treated and observed daily for two weeks (Figure 1).

### 2.7. Blood Sampling and Organ Collection

Twelve hours after the last dose of CCl_4_ injection, all animals were anesthetized by light ethyl ether inhalation and sacrificed. Blood samples were collected from the carotid arteries and centrifuged at 3000  rpm for 10 min under cool temperature (4°C) to separate the plasma. The separated plasma was stored at −20°C for further assessments. Besides, the liver was weighed and conserved for the preparation of the liver homogenate (10% w/v) in sodium phosphate buffer (pH 7.0) and stored at −20°C for biochemical analysis.

The liver index was calculated by the following formula [28]: liver index (%) = weight of liver/weight of body x 100%.

### 2.8. Biochemical Parameters Determination

The biochemical parameters such as serum enzymes: aminotransferases (AST and ALT) [29], alkaline phosphatase (ALP) [30], bilirubin (total and direct) [31], total cholesterol [32], triglycerides (TG) [33], high-density lipoprotein (HDL-c) [34], low-density lipoprotein (LDL-c), very low-density lipoprotein (VLDL-c), total protein (TP), glucose, urea, uric acid, and creatinine were evaluated by using an autoanalyzer (Architect c-Systems, Hamburg, Germany) by using a commercial kit. All analyses were performed in triplicate for every sample.

LDL-cholesterol was computed according to Friedewald et al., using the following equation: LDL-c = total cholesterol−[HDL-c + very low-density lipoprotein (VLDL-c)]. VLDL-c was calculated according to the formula as follows [35]: VLDL-c = triglycerides/5.

### 2.9. Determination of Malondialdehyde (MDA)

The concentration of liver lipid peroxidation was measured through the estimation of MDA by using thiobarbituric acid (TBA) [36]. In brief, 0.5 mL of TCA (30% w/v) was added to 0.5 mL of liver homogenate, and the mixture was centrifuged at 3500 rpm for 10 min at 4°C. 1 mL of the supernatant was added to 1 mL of TBA (0.67% w/v), and the mixture was placed in a boiling water bath for 10 min. The reaction mixture was stopped in an ice-cold bath. The absorbance of the solution was measured at 535 nm. The results were expressed in nanomoles of MDA produced per gram of tissue, using the following molar extinction coefficient: 1.56 × 10^5^ M^−1^cm^−1^.

### 2.10. Statistical Analysis

All values are expressed as mean ± SEM. The statistical differences among different groups were analyzed using one-way of analysis of variance (ANOVA), for determining the significant difference. The intergroup significance was analyzed using Turkey's post hoc test. The difference was considered significant if *p* < 0.05, moderately significant if *p* < 0.01, and highly significant if *p* < 0.001.

## 3. Results

### 3.1. Effect of AECe on the Liver Weight and Liver Index of Rats

**Figure 2:**
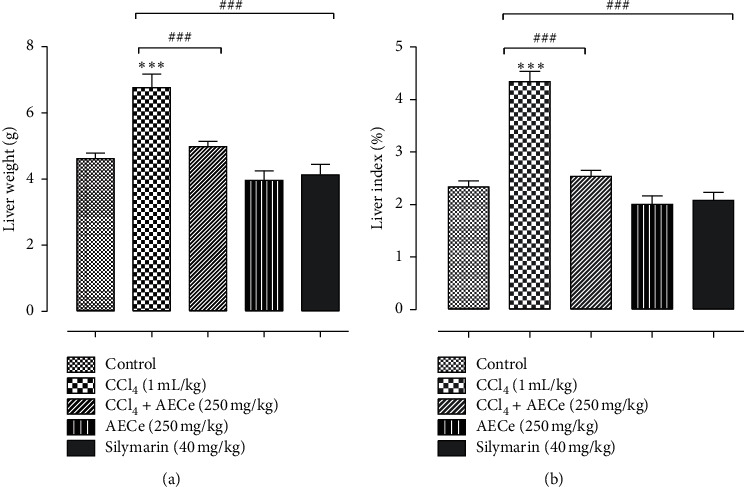
Effect of CCl_4_, AECe (250 mg/kg), and silymarin (40 mg/kg) on liver weight (a) and liver index (b). Values are mean ± SEM (*n* = 6) and analyzed with one-way ANOVA followed by Tukey's test.  ^*∗∗∗*^*p* < 0.001 vs. control group.  ^###^*p* < 0.001 vs. CCl_4_ group.

As shown in Figure 2, the normal rats treated with AECe (250 mg/kg) did not affect the liver weight and liver index compared with those of the normal control rats, indicating that the dose of AECe may have no liver toxicity in rats. After CCl_4_ administration, the liver weight and liver index significantly increased in rats (*p* < 0.001), indicating serious hepatomegaly that was markedly suppressed by a dose of AECe (250 mg/kg) and silymarin (*p* < 0.001 and *p* < 0.001*;* respectively).

### 3.2. Effect AECe on ALT, AST, and ALP

ALT, AST, and ALP are sensitive markers of the liver, and their elevated levels are indicative of liver damage. As shown in Table 1, no marked changes of AST, ALT, and ALP levels were detected in normal control rats and the AECe group, which confirmed the safety of AECe at a dose of 250 mg/kg. The injection of CCl_4_ to the rats induced liver injury, which represented markedly elevating activities of AST, ALT, and ALP serum levels compared with the normal control group. However, the AECe treatment (250 mg/kg) induced a significant (*p* < 0.05, *p* < 0.001) decrease in the CCl_4_-induced elevation of serum enzymes AST, ALT, and ALP compared to the CCl_4_-treated group.

The effect of AECe is comparable with that of the silymarin treatment. These results indicated a protective effect of AECe on CCl_4_-induced liver injury in rats.

### 3.3. Effect of AECe on Total and Direct Bilirubin

As shown in Table 1, the administration of CCl_4_ to the rats induced a significant (*p* < 0.001) increase in total and direct bilirubin levels, indicating the impaired excretory function of the liver. On the other hand, treatment with AECe at a dose of 250 mg/kg and silymarin (40 mg/kg) produced a highly significant (*p* < 0.01; *p* < 0.05) fall in the total and direct bilirubin levels compared to the CCl_4_-treated rats.

### 3.4. Effect of AECe on Total Protein

In CCl4 intoxicated rats, serum total protein level was decreased significantly (*p* < 0.001) when compared to the normal control group (Table 1). The oral administration of aqueous extract of *C. europaea* and silymarin reversed the depletion of total protein significantly (*p* < 0.001 and *p* < 0.001, respectively) when compared with CCl_4_-treated rats.

### 3.5. Effect of AECe on Lipid Peroxidation

**Figure 3:**
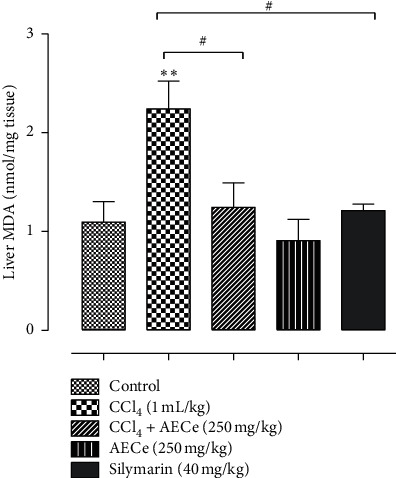
Effect of AECe (250 mg/kg) on lipid peroxidation in the liver of rats with CCl_4_-induced liver damage. The values represent the mean ± SEM, with *n* = 6.  ^*∗∗*^*p* < 0.01 vs. control group;  ^#^*p* < 0.05 vs. CCl_4_ group.

To evaluate the protective effect of aqueous extract of *C. europaea* against lipid peroxidation, MDA content was measured in liver homogenate (Figure 3). The administration of CCl_4_ alone induced a significant increase in MDA content (*p* < 0.01) compared with the normal control group. The doses of AECe (250 mg/kg) and silymarin (40 mg/kg) significantly suppressed the formation of MDA (*p* < 0.05) induced by CCl_4_ treatment.

### 3.6. Effect of AECe on Total Cholesterol, Triglycerides, VLDL-c LDL, HDL, and Plasma Glucose

The administration of CCl_4_ alone to the animals resulted in a marked increase in the plasma glucose, triglycerides, and VLDL levels (*p* < 0.001; *p* < 0.01 and *p* < 0.01, respectively) when compared to the normal control group. The rats treated with AECe (250 mg/kg) and the standard treatment silymarin (40 mg/kg), showed a significant reduction in all of the parameters that were increased in the CCl_4_-treated group. Overall, the results observed after administration of AECe at 250 mg/kg were comparable to those of silymarin at 40 mg/kg. On the other hand, no significant differences were detected in total cholesterol, LDL, and HDL levels in the CCl_4_-treated group compared to the normal control group (Table 2).

### 3.7. Effect of AECe on Creatinine, Urea, and Uric Acid

The plasma concentration of creatinine, urea, and uric acid was examined as biomarkers of renal function. As shown in Table 3, no significant differences in creatinine, urea, and uric acid levels were detected in the CCl_4_-treated group compared with the normal control group.

## 4. Discussion

Liver disease is a metabolic disorder, which is the most common cause of mortality and morbidity worldwide. Hence, medicinal herbs with hepatoprotective properties have received considerable attention from researchers. Recently, medicinal herbs have been utilized by researchers in experiments to investigate their hepatoprotective properties on animals [37]. In this study, we aimed to investigate the hepatoprotective effect of AECe on liver damage by measuring serum levels of aminotransferases (AST and ALT) activities, as enzyme markers of hepatocellular damage [38].

Liver injuries are induced by carbon tetrachloride in rats models. CCl_4_ is a commonly used model for the investigation of hepatoprotective activity on various experimental animals [39]. The liver damage caused by CCl_4_ is similar to that produced by viral hepatitis [40]. The elevated serum enzyme levels of AST, ALT, and ALP have been attributed to the damaged structural integrity of the liver because they are cytoplasmic in origin and are released into the blood after hepatic damage [27]. Our findings showed that AST, ALT, and ALP activities were increased in rats with the CCl_4_ treatment alone in comparison with the normal control group. This elevation in hepatic markers has been attributed to the cells damaged or cell membranes became leaky and they are released into the circulation [38, 41]. In contrast, a significant reduction in plasma activities of AST, ALT, and ALP was found in rats with AECe + CCl_4_ in comparison with the CCl_4_-treated group. This decrease in serum levels of transaminases activities is in agreement with the commonly accepted view that transaminases activities return to normal due to the stabilization of plasma membrane, as well as repair of hepatic tissue damages caused by CCl_4_ [1]. This finding suggests that AECe protected the liver tissue from CCl_4_-induced injury. Besides, AECe ameliorated the excretory function of the liver, and this effect was shown by suppressing the elevation of the bilirubin (total and direct) serum level. The level of total protein was reduced due to the stabilization of the endoplasmic reticulum leading to protein synthesis [42]. When animals are treated with CCl_4_, it is metabolically activated in the hepatic cell by cytochrome P450, generating a highly reactive carbon-centered Trichloromethyl radical. This radical reacts with oxygen to form the Trichloromethyl peroxyl radical, CCl_3_OO ^*∗*^ [43]. These two free radicals initiate the chain reaction of lipid peroxidation [6] and that is usually measured through its catabolite MDA [44]. The increase of MDA content in the liver of the CCl_4_-treated group was found in this study in good agreement with previous studies [45]. AECe decreased the MDA content in the liver significantly as compared to the CCl_4_-treated group. This is can be explained by the inhibition of lipid peroxidation and its propagation in the liver.

CCl_4_ brings an increase in plasma glucose levels in rats treated with CCl_4_ alone in comparison with the normal control group. This elevation may be due to the destruction of liver cells or disruption of glycogen storage due to the degradation of glycogen to glucose in hepatocytes after treatment with CCl_4_, which leads to increased plasma glucose concentration [46]. However, rats treated with AECe + CCl_4_ showed a significant reduction in plasma glucose concentration in comparison with the CCl_4_-treated group. In our investigation, AECe could enhance insulin secretion and stimulate the storage of glucose by the peripheral glucose uptake [19]. For the lipid profile, the serum levels of triglyceride and VLDL-c showed remarkable increases in CCl_4_-treated rats. Previous studies have indicated that increased VLDL is the result of disturbance of lipid metabolism induced by CCl_4_ intoxication. Triglycerides accumulation in the cytoplasm of hepatocytes leads to hepatic steatosis [1, 38]. However, treatment with AECe corrected this elevation. This effect indicates that the extract improved metabolic function by restoring serum triglycerides (TG) and VLDL levels to normal values compared to the CCl_4_-treated group. The other lesion of hepatic injury was hepatomegaly. While liver index was an objective indicator to reflect hepatomegaly, eliminating individual variation led to the difference of liver weights. In the present study, the liver index significantly enlarged in the CCl_4_-treated group, which indicated that CCl_4_ caused the hepatic damage and hepatomegaly. However, the treatment with AECe (250 mg/kg) restored the liver weight and the liver index to the condition almost like in the normal group. On the other hand, the study revealed that the biochemical parameters of the kidney did not show any variation (not significant) when compared to the normal control group. These results showed that CE has a significant hepatoprotective effect against carbon tetrachloride (CCl_4_). Free-radical production plays a key role in the mechanism pathway of CCl_4_-induced acute liver injury. Hence, the scavenging of free radicals is one of the major antioxidation mechanisms to inhibit the hepatotoxicity of CCl_4_ and reduce liver damage [47]. The characteristic phytochemical constituents in Caralluma species are glycosides, flavone glycoside, triterpenoids, flavonoids, tannins, alkaloids, and saponins. The phytoconstituents such as flavonoids, glycosides, triterpenoids, alkaloids, and saponins are known to possess hepatoprotective activity. Flavonoids have been known for their antioxidant and antiperoxidant properties leading to hepatoprotective activities [48]. Consequently, we suggest that the hepatoprotective activity of CE may be due to the presence of some of these components and/or other phytochemical compounds. However, further studies are required before we could conclude on the exact mechanism(s) involved in the hepatoprotective activity of the CE, and phytochemical studies are needed to isolate active compounds responsible for this activity.

## 5. Conclusions

Experimental evidence obtained in the present study showed that the oral administration of AECe exerted favorable hepatoprotective activity against carbon tetrachloride-induced liver damage. This activity may be due to the presence of flavonoids and other components present in the plant. However, complementary *in vitro* and *in vivo* studies will be necessary to confirm these findings and explore the mechanism responsible for this hepatoprotective effect.

## Figures and Tables

**Table 1:** Effect of aqueous extract of *Caralluma europaea* against carbon tetrachloride-induced hepatotoxicity-related parameters in rats.

Treatment (*n* = 6)	ALT (IU/L)	AST (IU/L)	ALP (IU/L)	Total bilirubin (mg/L)	Direct bilirubin (mg/L)	Total protein (g/L)
Normal control	36.33 ± 4.44	105.70 ± 9.7	171.33 ± 31.20	0.65 ± 0.11	1.00 ± 0.00	60.15 ± 0.93
CCl_4_ (1 mL/kg )	1014.33 ± 87.00 ^*∗∗∗*^	1189.83 ± 114.53 ^*∗∗∗*^	486.50 ± 34.84 ^*∗∗∗*^	4.45 ± 0.49 ^*∗∗∗*^	3.83 ± 0.47 ^*∗∗∗*^	38.71 ± 2.37 ^*∗∗∗*^
CCl_4_ + AECe (250 mg/kg)	668.20 ± 22.14 ^###^^Ns^	891.83 ± 32.33 ^#^^Ns^	245.33 ± 18.81 ^###^^Ns^	2.60 ± 0.26 ^*∗∗*^ ^##^^Ns^	2.50 ± 0.22 ^*∗∗*^ ^##^^**††**^	55.68 ± 1.17 ^###^^Ns^
AECe (250 mg/kg)	35.83 ± 5.52	97.83 ± 5.09	148.70 ± 17.53	0.75 ± 0.14	1.16 ± 0.16	60.06 ± 1.32
CCl_4_ + silymarin (40 mg/kg)	640.00 ± 39.83 ^*∗∗∗*^ ^###^	702.16 ± 55.59 ^*∗∗∗*^ ^###^	335.83 ± 23.53 ^*∗∗∗*^ ^##^	1.78 ± 0.20 ^###^	1.16 ± 0.16 ^###^	58.08 ± 1.54 ^###^

Values are expressed as mean ± SEM, (*n* = 6). Data were analyzed by one-way ANOVA followed by Tukey's test.  ^*∗∗*^*p* < 0.01 when compared to the normal control group;  ^*∗∗∗*^*p* < 0.001 when compared to the normal control group.  ^#^*p* < 0.05 when compared to the CCl_4_ group;  ^#^ ^#^*p* < 0.01 when compared to the CCl_4_ group;  ^###^*p* < 0.001 when compared to the CCl_4_ group. Ns = not significant when compared to the CCl_4_ + silymarin group; ^††^*p* < 0.01 when compared to the CCl_4_ + silymarin group.

**Table 2:** Effect of aqueous extract of *Caralluma europaea* on CCl_4_-induced lipid profile changes in rats.

Treatment (*n* = 6)	Cholesterol (g/L)	Triglycerides (g/L)	HDL (g/L)	LDL (g/L)	VLDL (g/L)	Glucose (g/L)
Normal control	0.59 ± 0.04	0.38 ± 0.03	0.23 ± 0.01	0.28 ± 0.02	0.07 ± 0.00	1.04 ± 0.03
CCl_4_ (1 mL/kg )	0.58 ± 0.01	0.64 ± 0.06 ^*∗∗*^	0.18 ± 0.00	0.26 ± 0.01	0.12 ± 0.01 ^*∗∗*^	1.57 ± 0.06 ^*∗∗∗*^
CCl_4_ + AECe (250 mg/kg)	0.54 ± 0.03 Ns	0.31 ± 0.02 ^###^^Ns^	0.21 ± 0.01^Ns^	0.26 ± 0.04 **Ns**	0.06 ± 0.00 ^###^^Ns^	1.33 ± 0.04 ^*∗∗*^ ^##^^Ns^
AECe (250 mg/kg)	0.52 ± 0.04	0.30 ± 0.02	0.22 ± 0.01	0.23 ± 0.04	0.06 ± 0.00	1.07 ± 0.06
CCl_4_ + silymarin (40 mg/kg)	0.55 ± 0.03	0.34 ± 0.01 ^###^	0.24 ± 0.01	0.21 ± 0.03	0.05 ± 0.01 ^###^	1.22 ± 0.05 ^###^

Values are expressed as mean ± SEM, (*n* = 6). Data were analyzed by one-way ANOVA followed by Tukey's test.  ^*∗∗*^*p* < 0.01 when compared to the normal control group;  ^*∗∗∗*^*p* < 0.001 when compared to the normal control group.  ^##^*p* < 0.01 when compared to the CCl_4_ group;  ^###^*p* < 0.001 when compared to the CCl_4_ group. Ns = not significant when compared to the CCl_4_ + silymarin group.

**Table 3:** Effect of aqueous extract of *Caralluma europaea* on CCl_4_-induced kidney function test in serum.

Treatment (*n* = 6)	Creatinin (mg/mL)	Urea (g/L)	Uric acid (mg/L)
Normal control	5.763 ± 0.13	0.593 ± 0.03	11.883 ± 1.46
CCl4 only (1 mL/kg )	5.857 ± 0.24^ns^	0.648 ± 0.05^ns^	9.600 ± 1.04^ns^
AECe (250 mg/kg) + CCl4	5.737 ± 0.11^ns^	0.521 ± 0,05^ns^	8.467 ± 0.46^ns^
AECe only (250 mg/kg)	5.941 ± 0.23^ns^	0.671 ± 0.04	11.633 ± 0.98
CCl4 + silymarin (40 mg/kg)	5.757 ± 0.11	0.558 ± 0.02^ns^	10.180 ± 0.27^ns^

Data expressed in mean ± SEM (*n* = 6) and analyzed with one-way ANOVA followed by Tukey's test. The meaning was compared with the normal control group. ^ns^ = not significant.

## Data Availability

Data used to support the findings of this study are available upon request.

## Supplementary Materials

Supplementary Materials Table 1: *effect of aqueous extract of Caralluma europaea against carbon tetrachloride-induced* hepatotoxicity-related parameters in rats. Values are expressed as mean ± SEM (*n* = 6). Data were analyzed by one-way ANOVA followed by Tukey's test. *p*^*∗∗*^ < 0.01 when compared to the normal control group; *p*^*∗∗∗*^ < 0.001 when compared to the normal control group. *p*^#^ < 0.05 when compared to the CCl_4_ group; *p*^##^ < 0.01 when compared to the CCl_4_ group; and *p*^###^ < 0.001 when compared to the CCl_4_ group. Ns = not significant when compared to CCl_4_ + silymarin group; *p*^††^ <0.01 when compared to CCl_4_ + silymarin group.
