# Supplementary material for: Evaluation of Hepatoprotective Activity of Caralluma europaea Stem Extract against CCl4-Induced Hepatic Damage in Wistar Rats
Source: Adv Pharmacol Pharm Sci. 2021 Jan 7;2021:8883040. doi: 10.1155/2021/8883040 (PMC7810557; doi:10.1155/2021/8883040)
Supplement: Supplementary Materials — Table 1: effect of aqueous extract of Caralluma europaea against carbon tetrachloride-induced hepatotoxicity-related parameters in rats. Values are expressed as mean ± SEM (n = 6). Data were analyzed by one-way ANOVA followed by Tukey's test. p∗∗ < 0.01 when compared to the normal control group; p∗∗∗ < 0.001 when compared to the normal control group. p# < 0.05 when compared to the CCl4 group; p## < 0.01 when compared to the CCl4 group; and p### < 0.001 when compared to the CCl4 group. Ns = not significant when compared to CCl4 + silymarin group; p†† <0.01 when compared to CCl4 + silymarin group. [file 8883040.f1.docx]

**Tableau 1:** Effect of aqueous extract of *Caralluma europaea* against carbon tetrachloride-induced hepatotoxicity-related parameters in rats.

Values are expressed as mean ± SEM, (n=6). Data were analyzed by one-way ANOVA followed by turkey’s test. *p*** < 0.01 when compared to normal control group; *p**** < 0.001 when compared to normal control group.

*p*^#^ < 0.05 when compared to CCl_4_ group; *p*^##^ < 0.01 when compared to CCl_4_ group; *p*^###^ < 0.001 when compared to CCl_4_ group.

Ns = not significant when compared to CCl_4_ + Silymarin group; *p*†† < 0.01 when compared to CCl_4_ + Silymarin group.
